# A Proteomic Study of Memory After Imprinting in the Domestic Chick

**DOI:** 10.3389/fnbeh.2015.00319

**Published:** 2015-11-26

**Authors:** Maia Meparishvili, Maia Nozadze, Giorgi Margvelani, Brian J. McCabe, Revaz O. Solomonia

**Affiliations:** ^1^School of Natural Sciences and Engineering, Institute of Chemical Biology, Ilia State UniversityTbilisi, Georgia; ^2^I. Beritashvili Institute of Experimental BiomedicineTbilisi, Georgia; ^3^Department of Zoology, Sub-Department of Animal Behavior, University of CambridgeCambridge, UK

**Keywords:** learning, recognition memory, IMM, IMHV, membrane and mitochondrial proteins, cognin, dynamin

## Abstract

The intermediate and medial mesopallium (IMM) of the domestic chick forebrain has previously been shown to be a memory system for visual imprinting. Learning-related changes occur in certain plasma membrane and mitochondrial proteins in the IMM. Two-dimensional gel electrophoresis/mass spectrometry has been employed to identify more comprehensively learning-related expression of proteins in the membrane-mitochondrial fraction of the IMM 24 h after training. We inquired whether amounts of these proteins in the IMM and a control region (posterior pole of the nidopallium, PPN) are correlated with a behavioral estimate of memory for the imprinting stimulus. Learning-related increases in amounts of the following proteins were found in the left IMM, but not the right IMM or the left or right PPN: (i) membrane cognin; (ii) a protein resembling the P32 subunit of splicing factor SF2; (iii) voltage-dependent anionic channel-1; (iv) dynamin-1; (v) heterogeneous nuclear ribonucleoprotein A2/B1. Learning-related increases in some transcription factors involved in mitochondrial biogenesis were also found, without significant change in mitochondrial DNA copy number. The results indicate that the molecular processes involved in learning and memory underlying imprinting include protein stabilization, increased mRNA trafficking, synaptic vesicle recycling, and specific changes in the mitochondrial proteome.

## Introduction

Visually naïve domestic chicks come to recognize a visual stimulus by being exposed to it and subsequently approach that stimulus in preference to other stimuli. This learning process is known as visual imprinting and involves the formation of a memory of the imprinting stimulus (for review, see e.g., Bolhuis, 1991). Imprinting is a powerful and rapid form of learning, is readily observed in many precocial species and is associated with processes having the characteristics of recognition memory observed in a wide range of animals (Bateson, 1990).

Visual imprinting in the domestic chick offers a powerful means of investigating experimentally the molecular and neural basis of memory. The advantages of visual imprinting for the study of memory are:- (i) A restricted region within the chick forebrain, the intermediate and medial mesopallium (IMM) has been identified as being of crucial importance for visual imprinting. The available evidence (cf. Horn, 1985, 2004; McCabe, 2013) indicates that storage of information about the imprinting stimulus occurs in the IMM. This region was formerly known as the intermediate and medial hyperstriatum ventrale (IMHV) (Reiner et al., 2004). (ii) The visual experience of the recently-hatched chick is minimal and can be controlled, giving a low baseline against which learning-related changes in the brain may be detected and analyzed. (iii) Chicks do not require food or water for the early part of the 3- to 4-day sensitive period for imprinting and thus avoid a complication inherent in animals that must be fed. (iv) Newly-hatched chicks are very active, vocalize extensively and offer abundant behavioral read-out from which learning and memory may be inferred (Horn, 1985, 2004; McCabe, 2013; Solomonia and McCabe, 2015).

By exploiting the above advantages it has been possible to demonstrate a series of learning-related molecular changes in the IMM, which are involved in a progression from transient/labile to trophic synaptic modifications, culminating in stable recognition memory (for reviews see McCabe, 2013; Solomonia and McCabe, 2015).

Some of these changes are in mitochondrial and plasma membrane proteins, most of which are observed 24 h after training (Solomonia et al., 1997, 1998, 2000, 2003, 2005, 2008, 2011, 2013). Levels of the mitochondrial proteins studied (subunits CO-I and CO-II of cytochrome c oxidase, of critical importance for oxidative metabolism) were found to be constitutively highly correlated with one another in the left IMM, giving rise to the hypothesis that this region is especially adapted for learning just after hatching, perhaps through precocious development (Solomonia et al., 2011). The ultimate aim of our experiments is to identify the fine molecular signature of memory formation in imprinting. The specific aims of the present study were twofold. First, in order to study learning-related changes in proteins more comprehensively, we adopted a systematic, proteomic approach. Two-dimensional (2-D) gel electrophoresis and mass-spectrometry (MS) were used to identify proteins whose levels in the IMM were changed 24 h after learning and which therefore may have contributed to memory formation. The roles in imprinting of candidate proteins thus identified were then investigated in detail using immunoblotting. Second, we have further investigated the role of mitochondria in the IMM by measuring copy number of mitochondrial DNA and levels of proteins associated with mitochondrial biogenesis. Because many of the learning-related changes in the IMM are biased toward (or restricted to) the left IMM (cf. McCabe, 1991, 2013), and because the left IMM is characterized by a highly significant correlation between mitochondrial proteins CO-I and CO-II (Solomonia et al., 2011), we have paid particular attention to the possibility of lateralization.

## Materials and methods

### Behavioral training and testing

Fertile eggs (*Gallus gallus domesticus*), of the same genetic background (Cobb500 strain) were obtained from a local supplier in Tbilisi. Chicks were hatched, reared and trained as described previously (Solomonia et al., 2005). In total, 48 batches of chicks were hatched and reared in darkness. Each batch comprised up to three trained chicks and an untrained control chick, all from the same hatch. When 22–28 h old, the chicks to be trained were placed individually in running wheels and exposed to a visual imprinting (training) stimulus (a rotating, internally illuminated red box; for details see Bolhuis et al., 2000). The maternal call of a hen was played during this training procedure. As a chick attempted to approach the training stimulus, it rotated the running wheel; revolutions of the wheel (circumference 94 cm) were counted to provide a measure of approach activity (“training approach”). Chicks that have become imprinted have learned the characteristics of the training stimulus and subsequently prefer it to an alternative visual stimulus (Sluckin, 1972). Ten minutes after training, each chick was given a preference test (McCabe et al., 1981). In this test the chick was shown, sequentially, either the training stimulus or an alternative stimulus—a rotating, internally illuminated blue cylinder (Bolhuis et al., 2000). The maternal call was not played during the preference test. A preference score was then calculated to provide a measure of each chick's preference and hence of the strength of learning. The preference score was calculated as follows:

$$\text{Preference score}=\frac{\text{1}00\;\times \;\left(\text{approach to training stimulus}\right)}{\begin{array}{l} (\text{approach to training stimulus }\\ \;+\text{ approach to alternative stimulus}) \end{array}}.$$

If both stimuli are approached equally, the preference score is 50 (no choice, indicative of no learning). If the chick approaches only the training stimulus, the preference score is 100, indicating strong learning. There are individual differences in chicks' preference scores after a fixed period of training. This variation was used to relate changes in total amounts of protein and mitochondrial DNA to preference score (Horn and Johnson, 1989; cf. McCabe and Horn, 1988). The trained chicks achieved a range of preference scores: where possible, one chick was selected with a preference score >40 and ≤60 (poor learner); one with a preference score >60 and ≤80 (intermediate learner) and one with preference score >80 (good learner). In addition, there was one untrained chick in each batch.

The chicks were sacrificed 24 h after the end of training. Four pieces of tissue were removed, from the left and right IMM and from the left and right PPN. The locations of the IMM and PPN are shown in a previous publication (Solomonia et al., 2013, Figures 1A,B). For details of the methods of removal of tissue from the IMM and PPN, see Horn (1991) and Solomonia et al. (1998), respectively. After removal, each piece of tissue (a sample) was immediately covered in dry ice. Thus, in each batch there were four samples from each of up to four chicks (one untrained, up to three trained), yielding up to 16 samples in all. Samples were coded after collection and all further procedures were conducted blind. All behavioral experiments were carried out at the I. Beritashvili Centre of Experimental Biomedicine according to the requirements of the Institute Bioethics Committee. The number of animals used was estimated on past experience to be the minimum required for adequate statistical analysis.

**Figure 1:**
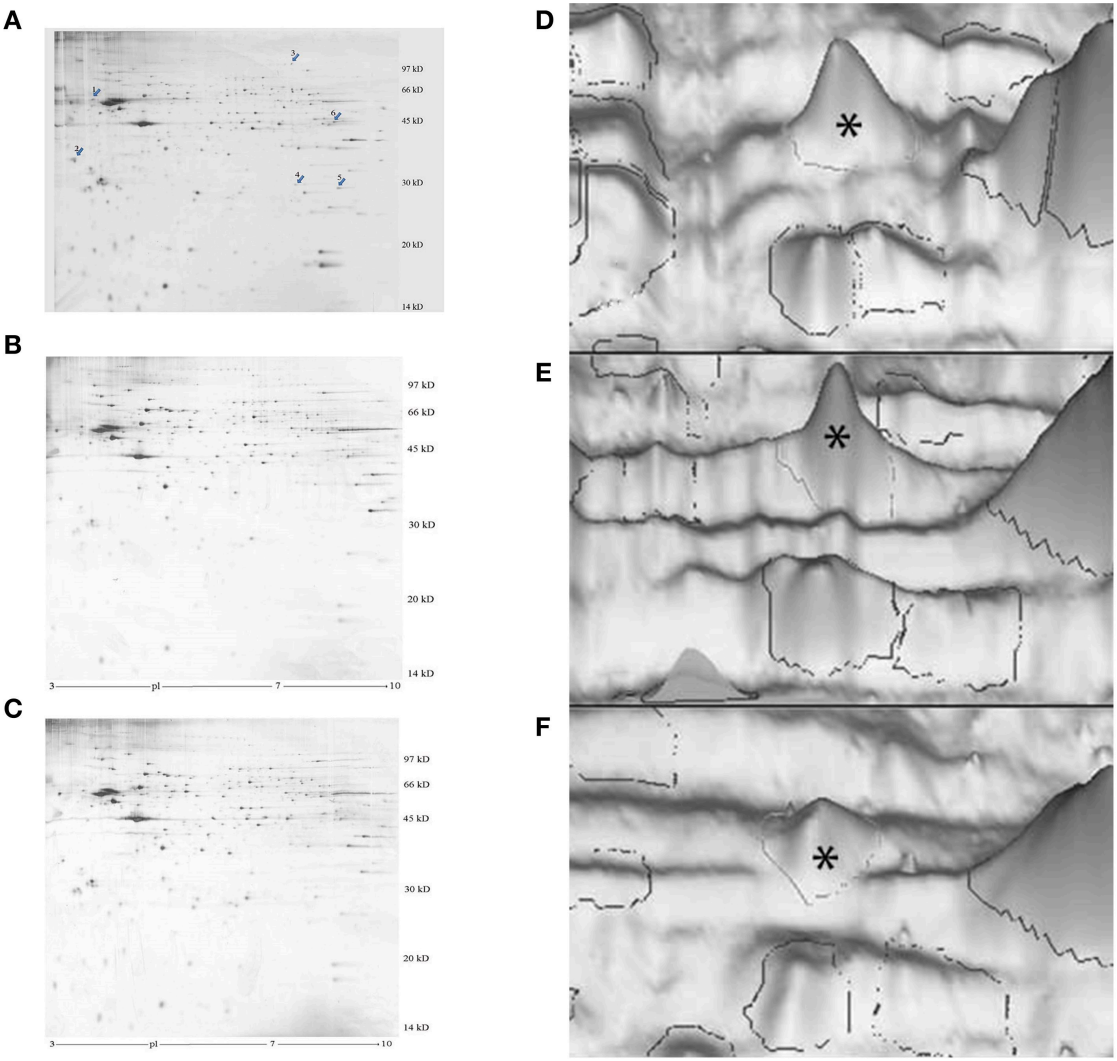
**Representative images of silver-stained 2-D electrophoresis gels of the P2 membrane-mitochondrial fraction from the left IMM. (A–C), photographs of gels**. The arrows in **(A)** indicate significantly changed proteins: 1, cognin; 2, M-P38; 3, dynamin-1; 4, VDAC-1; 5, hnRNP A2/B1. A good learner, **(B)** poor learner, **(C)** untrained chick. **(D–F)**, 3-D representations, peak for M-cognin indicated by asterisk. **(A)** good learner, **(B)** poor learner, **(C)** untrained chick.

### Tissue fractionation for 1-dimensional electrophoresis and immunoblotting

Samples were rapidly homogenized in 20 mM Tris-HCl (pH 7.4), 0.32 M sucrose, 1 mM ethylendiamintetraacetic acid, 1 mM sodium orthovanadate, 10 mM sodium pyrophosphate, 0.5 mM ethylene glycol-bis(2-aminoethylether)-N,N,N',N'-tetraacetic acid, and a cocktail of protease inhibitors (Sigma, P8340). One-third of the whole homogenate was saved for the determination of nuclear transcription factors involved in mitochondrial biogenesis (see below) and the remaining two-thirds centrifuged at 1000 g for 10 min. The supernatant was further centrifuged at 15,000 g for 20 min. The resulting supernatant is referred to as the cytoplasmic fraction. The pellet was washed once and is referred to as the P2 mitochondrial-membrane fraction. A concentrated solution of sodium dodecyl sulfate (SDS) was added to the whole homogenate and cytoplasmic fractions to give a final concentration of 5%. The P2 mitochondrial-membrane fraction was also dissolved in 5% SDS. Amounts of nuclear respiratory factor 1 (NRF-1) and peroxisome proliferator activated receptor gamma coactivator-1-α (PGC1-α) were measured in total homogenate fractions. Amounts of cognin and P38 were measured in both cytoplasmic and P2 mitochondrial-membrane fractions. Amounts of dynamin, voltage-dependent anion channel 1 (VDAC-1), heterogeneous nuclear ribonucleoprotein A2/B1 (hnRNP A2/B1), and mitochondrial transcription factor A (MTFA) were measured in P2 mitochondrial-membrane fractions.

Protein concentrations in homogenate, cytoplasmic and P2 mitochondrial-membrane fractions were determined in quadruplicate using a micro bicinchoninic acid protein assay kit (Pierce). Aliquots containing 30 μg of protein in 30 μl were subjected to SDS gel electrophoresis and Western blotting (Solomonia et al., 2003). After protein had been transferred onto nitrocellulose membranes, the membranes were stained with Ponceau S solution to confirm transfer and uniform gel loading. The nitrocellulose membranes were stained with the following commercially available primary antibodies: 1. anti-P 32 polyclonal antibody (AB2991, Millipore); 2. anti-dynamin-1 monoclonal antibody (ab14448, Abcam); 3. anti-VDAC-1/porin polyclonal antibodies (ab15895, Abcam); 4. Anti-hnRNP A2/B1 monoclonal antibody (ab6102 Abcam); 5. Anti-MTFA polyclonal antibody (ab69295, Abcam); 6. Anti-NRF-1 polyclonal antibody (ab86516, Abcam); 7. Anti-PGC1-α+β polyclonal antibody (ab72230, Abcam). The control peptide ab73600 was used to identify the α-isoform of PGC1. Rabbit polyclonal antibodies against chicken cognin (P09102, UniProt) were produced against the 21mer peptide DDDLEDLETDEETDLEEGDDD, which contained a terminal cysteine for conjugation to a carrier protein. Antibodies were purified on an antigen-affinity column. The specificity of antibody reaction was confirmed by adsorption of control peptide.

**Figure 2:**
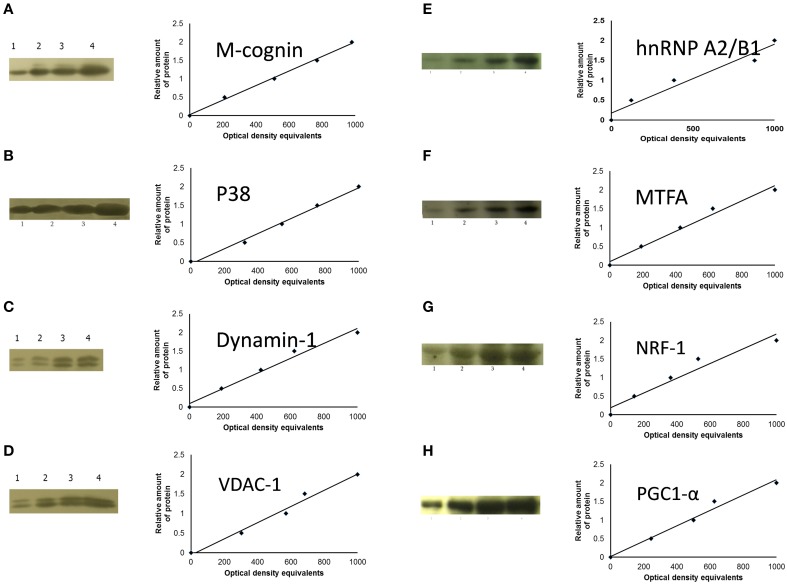
**Sample films and calibration plots for: (A), M-cognin; (B), M-P38; (C), dynamin-1; (D), VDAC-1; (E), hnRNP A2/B1; (F), MTFA; (G), NRF-1; (H), PGC1-α**. Left-hand columns, sample radiographs; right-hand columns, calibration plots (lines fitted by linear least-squares regression).

Where molecular weights of the proteins were sufficiently disparate, the nitrocellulose membranes were cut into 2–3 parts and stained with different antibodies. Standard immunochemical procedures were performed using peroxidase-labeled secondary antibodies and SuperSignal West Pico Chemiluminescent substrate (Pierce). Blots were then exposed with intensifying screens to X-ray films pre-flashed with Sensitize (Amersham). Optical density of protein bands was measured using LabWorks 4.0 (UVP) software. Autoradiographs were calibrated using standard amounts of protein obtained either from total homogenate, cytoplasmic or P2 membrane fractions of the IMMs of a group of untrained chicks. Four standards (15, 30, 45, and 60 μg total protein) were applied to each gel. For these standards the optical densities of bands immunostained for the corresponding protein (e.g., cognin, VDAC-1, etc.) were plotted against amount of protein; in all these standards, least squares regression showed a significant fit to a straight line (see Figure 2). To obtain data for regression analysis optical density of each band from each sample was divided by the optical density which, from the calibration of the same autoradiograph, corresponded to 30 μg of total protein in the standard (Solomonia et al., 2000). This quantity is termed “relative amount of protein.” Variability attributable to differences between batches was removed by dividing relative amount of protein by the mean for that batch. Data expressed in this way will be referred to as “standardized relative amount” of protein.

For the immunoblotting experiments, samples were obtained from a total of 69 trained chicks and 32 untrained chicks in 32 batches; some data, identified as artifactual under blind conditions, were discarded. Data from experimental stained bands were not normalized with respect to actin or any other housekeeping protein because it cannot be guaranteed that such proteins are unaffected by imprinting (see also Dittmer and Dittmer, 2006; Li and Shen, 2013; Ghosh et al., 2014; Chen and Xu, 2015 for discussion of the unreliability of normalization to housekeeping proteins). Instead, we have controlled loading by Ponceau S staining, calibrated with protein standards and standardized using mean amount of protein in each batch (see above).

### 2-D electrophoresis

#### Tissue fractionation

The P2 mitochondrial-membrane fraction was obtained as described above. The pellet was dissolved in a sample buffer containing the final concentrations of the following components: 7 M urea, 2 M thiourea, 2% CHAPS, 2% triton X-100, 0.1% ASB-14, 2-mercaptoethanol, *2%* pharmalyte 3–10, bromophenol blue. Protein concentrations were determined in quadruplicate using a micro bicinchoninic acid protein assay kit (Pierce).

#### Isoelectric focusing

Strips (linear pH 3.0–10.0, 18 cm) were rehydrated overnight in the following solution: 8 M Urea, 0.5% triton X-100, 0.5% pharmalyte 3–10, 14 mM dithiothreitol (DTT). Isoelectric focusing was carried out with the following regime: 500 V for 3 h and 3500 V for 17.7 h. Forty micrograms of protein were loaded onto each strip.

#### Equilibration

Strips were equilibrated for 15 min in buffer comprising 0.05 M Tris-HCl (pH 6.8), 6 M urea, 30% glycerol, 3% SDS and 1% DTT and for the following 15 min in a buffer of the same composition except that it contained 2.5% iodoacetamide instead of 2.5% DTT.

#### SDS electrophoresis

SDS electrophoresis was run on 1 mm thick 12.5% polyacrylamide gels at 25°C with two steps: (i) 1–10 mA/gel, 80 V for 1 h and (ii) 12 mA/gel, 150 V for 17 h.

#### Staining, scanning and analysis

The gels were stained with silver without a glutaraldehyde step. Silver-stained gels were scanned with an Image scanner III Labscan 6.0. Images were digitized and processed using ImageMaster 2-D platinum 7.0 software. Six gels were run in parallel on a 2-D electrophoresis system (GE Healthcare). The P2 fractions from the left IMM were analyzed from chicks with high preference score (>85%, good learners) side-by-side with fractions from chicks with low preference score (< 65%, poor learners) and from untrained chicks. There were 6 such experiments (12 samples of left IMMs from trained and 6 samples of left IMMs from untrained chicks).

In each series of experiments those protein spots were selected which exhibited at least a 2-fold difference between good learners and untrained chicks or between good learners and poor learners. The data for the spots coinciding by location (pI and Molecular weight) from different experiments were analyzed by two-tailed *t*-test for significant differences between the groups; significance level was set at 5%. Significantly differentially expressed spots were cut out, de-stained and kept at < –20°C until MS analysis.

### In-gel digestion and MS analysis

Differentially expressed bands were cut from the gels. The in-gel digestion, mass spectrometry and analysis of peptide sequences were performed in the Cambridge Centre for Proteomics of the University of Cambridge.

#### Sample preparation

2-D gel spots were excised from the gel and transferred into a 96-well PCR plate. The spots were destained, reduced (DTT) and alkylated (iodoacetamide) and subjected to enzymatic digestion with trypsin overnight at 37°C. After digestion, the supernatant was pipetted into a sample vial and loaded into an autosampler for automated LC-MS/MS analysis.

#### LC-MS/MS experiments

Mass spectrometry experiments were performed using either LTQ linear ion trap (first series of MS experiments, identification of first two candidate proteins, see Results) or LTQ Orbitrap Velos instruments (second series of MS experiments, identification of another three candidate proteins, see Results) fitted with nanospray ion sources (ThermoFisher, Waltham, MA).

For the LTQ experiments, the separation of peptides was performed by reverse-phase chromatography using an Eksigent Ultra (Eksigent Technologies, Dublin, CA) HPLC pump at a flow rate of 300 nL/min and an LC-Packings (Dionex, Sunnyvale, CA) PepMap 100 column (C18, 75 μM i.d. × 150 mm, 3 μM particle size). Peptides were loaded onto an LC-Packings precolumn (Acclaim PepMap 100 C18, 5 μM particle size, 100A, 300 μM i.d × 5 mm) from the autosampler using 0.1% formic acid for 5 min at a flow rate of 5 μL/min to desalt samples and focus peptides prior to analytical separation. After this period, the six port valve was switched to allow elution of peptides from the precolumn onto the analytical column. Solvent A was water + 0.1% formic acid in water and solvent B was 5% acetonitrile + 0.1% formic acid in water. The gradient employed was 5–55% B in 40 min (total run time of 60 min). The LTQ instrument was operated in a data-dependent manner, in which a survey scan was performed to analyse the *m/z* values of ions which were eluted from a reverse-phase HPLC column. If ions were detected above a certain threshold as having a charge state of 2+ or 3+, they were automatically isolated, fragmented by CID and an MS/MS spectrum was generated.

For the Orbitrap Velos experiments, peptides were separated using an Eksigent NanoLC-1D Plus (Eksigent Technologies, Dublin, CA) HPLC system by reverse-phase chromatography at a flow rate of 300 nL/min through an LC-Packings (Dionex, Sunnyvale, CA) PepMap 100 column (C18, 75 μM i.d. × 150 mm, 3 μM particle size). Peptides were initially loaded onto a precolumn (Dionex Acclaim PepMap 100 C18, 5 μM particle size, 100 A, 300 μM i.d × 5 mm) from the autosampler with 0.1% formic acid for 5 min at a flow rate of 10 μL/min. After this period, the valve was switched to allow elution of peptides from the precolumn onto the analytical column. Solvent A was water + 0.1% formic acid and solvent B was acetonitrile + 0.1% formic acid. The gradient employed was 5–50% B in 30 min (40 min total run time). The LC eluant was sprayed into the mass spectrometer by means of a New Objective nanospray source. All *m/z* values of eluting ions were measured in an Orbitrap Velos mass analyzer, set at a resolution of 30,000. Data dependent scans (Top 20) were employed to automatically isolate and generate fragment ions by collision-induced dissociation in the linear ion trap, resulting in the generation of MS/MS spectra. Ions with charge states of 2+ to 4+ were selected for fragmentation.

#### Database searching

Post-run, the data were processed using Bioworks Browser (version 3.3.1 SP1, ThermoFisher). Briefly, all MS/MS spectra were converted to DTA (text) files using the Sequest Batch Search tool (within Bioworks). The DTA files were converted to a single file using a SSH script in the SSH Secure Shell Client program (Version 3.2.9 Build 283, SSH Communications Corp.). These combined files were then submitted to the Mascot search algorithm (Matrix Science, London UK) and searched against the NCBI *Gallus gallus* REFSEQ_082010 database (19127 sequences; 7388293 residues), using a fixed modification of carbamidomethyl and a variable modification of oxidation (M) and a significance threshold value of *p* < 0.05. A peptide cut-off score of 20 was also applied. For the LTQ data, a peptide mass tolerance of 1 Da and fragment ion mass tolerance of 0.8 Da were applied. For the Orbitrap data, the peptide and fragment mass tolerances were set to 25 ppm and 0.8 Da, respectively. The maximum number of missed cleavages was set to 2.

Additional mass spectrometry data are given in Supplementary Material.

### Mitochondrial DNA

The experiment to study mitochondrial DNA was conducted on 20 trained and 10 untrained chicks (10 batches). The DNA from chick brain tissue samples (left and right IMM, left and right PN from chicks with different preference scores and from untrained animals, see above) was isolated with a DNeasy Blood and Tissue Kit (Qiagen, Cat. No. 69504) and concentration measured by absorbance at wavelength 280/260 nm on Nanodrop. Relative mitochondrial DNA copy number was determined by real-time PCR using the Step One Plus Real-Time PCR System (Applied Biosystems) with the SYBR Green detection method. The part of the chicken mitochondrial DNA encompassing the 30 end of the NADH dehydrogenase subunit 5 (ND5) and the 50 end of cytochrome B was amplified (López-Andreo et al., 2005) and was normalized to that of the β-actin gene in nuclear DNA. For mitochondrial DNA the following primers were used: forward TCGCCCTCACAATCCTTACAA, reverse CTGGGAGGTCGATTAGGGAGT. For the beta actin, forward CAGACATCAGGGTGTGATGGTTGG, reverse GGGTGTTGAAGGTCTCAAACATG were used. The comparative C_T_ (ΔΔC_T_) method was used to determine the relative target quantity in samples (Livak and Schmittgen, 2001). The same fraction of mitochondrial DNA isolated from the IMMs of untrained chicks was used in all experiments as a reference sample. Amplicons from randomly chosen samples were sequenced at the Genomic Center of the National Center for Disease Control, Tbilisi, Georgia.

### Statistical analysis of immunoblotting and DNA data

Data from IMM and PPN were analyzed separately because only the IMM has been firmly implicated the IMM in memory (Horn, 1985). Statistical analysis was performed using R (R Core Team, 2015). A linear mixed model was fitted to standardized relative amount, with model terms Preference Score and Side, with Chick and Batch as random factors, Chick nested within Batch, in a split-plot design. The model was used to identify significant associations between substance amount and preference score, and regression coefficient (slope of fitted relationship between standardized relative amount and Preference Score). Regressions were also run separately for left and right sides of IMM PPN because learning-related effects found previously in IMM have often show hemispheric asymmetry; when such asymmetry occurs, even without a significant interaction between Preference Score and Side model terms, the effect has been found to predominate in left IMM (Solomonia and McCabe, 2015). By analyzing data separately on the two sides, we could evaluate the consistency of this pattern of asymmetry.

Regressions between relative amount and preference score are plotted in **Figures 4–7**. Standardized relative amount is plotted against preference score, together with the least-squares regression line. This line has been interpolated to the “no preference” score of 50, indicating no learning despite chicks' exposure to the training stimulus. The amount corresponding to preference score 50 is shown on the ordinate, with its standard error. This corresponding amount (intercept) is compared with the mean for untrained chicks by analysis of variance. If there is a significant regression with Preference Score, but the intercept at Preference Score 50 is statistically homogeneous with the mean untrained value, the implication is that influences other than learning (side-effects such as placement in running wheel, exposure to training stimulus, locomotor activity, stress, etc.) have had no significant effect. However, the significant regression with Preference Score indicates an association with learning.

For significant regressions, mean untrained value was also compared, by analysis of variance, with the standardized relative amount of protein (y-intercept) corresponding to the maximum preference score in that experiment. We thus enquired whether strong learning, estimated by interpolating the regression line to maximum preference score, was sufficient to change amount of protein from the control, untrained value.

To understand significant regressions further, residual variance from each significant regression (trained chicks) was compared with the residual variance in untrained chicks. This was to evaluate evidence that changes in amount were attributable either to a predisposition or to learning during training (cf. Horn and Johnson, 1989; McCabe, 2013). A significant regression might be due to a predisposition. That is, chicks hatched with very high (or, for negative regressions, very low) levels of the substance in question, may be predisposed to learn well when trained. If so, a significant regression need not be a consequence of training: data from the trained chicks may simply be a sample from the same population as untrained chicks, significantly associated with preference score because of chicks' predisposition to learn well. If so, the significant regression should result in a significantly smaller residual variance relative to the untrained value: the regression would have accounted for some variance and the residual variance would be correspondingly smaller. In contrast, if the residual variance from the regression were not significantly lower than the untrained variance, the predisposition hypothesis would not be supported. Rather, the data would indicate that the significant regression on preference score is a consequence of learning during the training period.

Regressions were taken to be learning-related if (i) regression with preference score was significant; (ii) intercept at preference score 50 was not significantly different from the untrained value; (iii) intercept at the highest preference score for that experiment was significantly different from the untrained value; (iv) residual variance from the regression was not significantly lower than the residual variance in untrained chicks.

## Results

### 2-D electrophoresis and mass spectrometry (MS)

The 2-D electrophoresis of P2 membrane-mitochondrial fractions revealed several protein bands with significant differences between good learners, poor learners and untrained chicks (Figure 1). By MS analysis bands were identified with the following proteins:-

1. Cognin (prolyl-4-hydroxylase/protein disulfide isomerase, accession number AAA49054.2).
2. Chicken protein accession number XP_415748.2, similar to p32 subunit of human splicing factor SF2 (referred to below as P38).
3. Voltage-dependent anionic channel-1, also known as porin-1, accession number NP_001029041.1 (referred to below as VDAC-1).
4. A protein similar to dynamin 1 isoform 1, accession number XP_001233250.1 (referred to below as dynamin-1).
5. Heterogeneous ribonucleoprotein A2/B1, accession number NP_001026156.1 (referred to below as hnRNP A2/B1).

### Results of western immunoblotting

**Figure 3:**
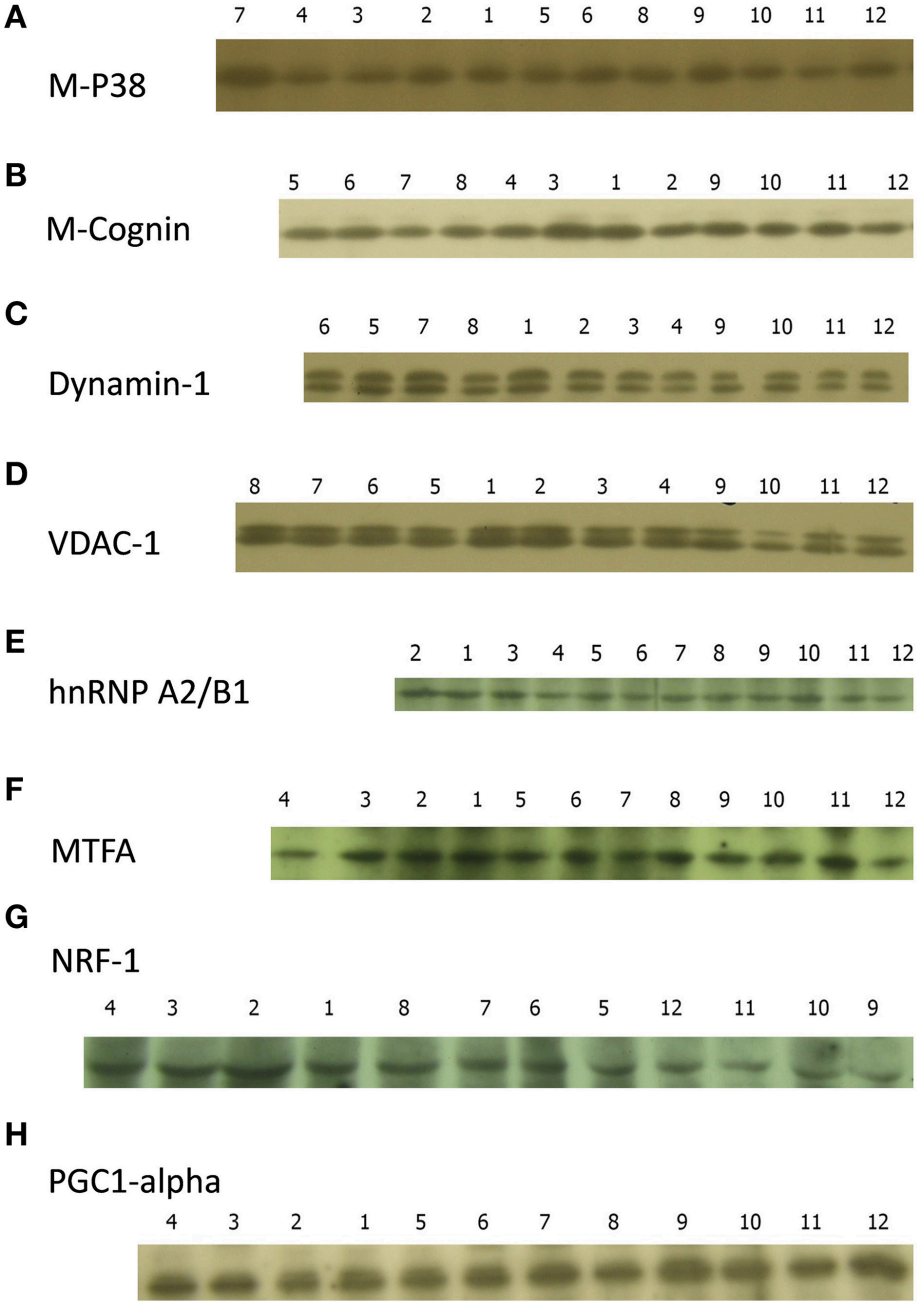
**Sample films for (A), M-cognin; (B), P38; (C), dynamin-1; (D), VDAC-1; (E), hnRNP A2/B1; (F), MTFA; (G), NRF-1; (H), PGC1-α**. Experimental conditions: 1 Good learner, left IMM; 2 Good learner, right IMM; 3 Good learner, left PPN; 4 Good learner, right PPN; 5 Poor learner, left IMM; 6 Poor learner, right IMM; 7 Poor learner, left PPN; 8 Poor learner, right PPN; 9 Untrained, left IMM; 10 Untrained, right IMM; 11 Untrained, left PPN; 12 Untrained, right PPN.

**Figure 4:**
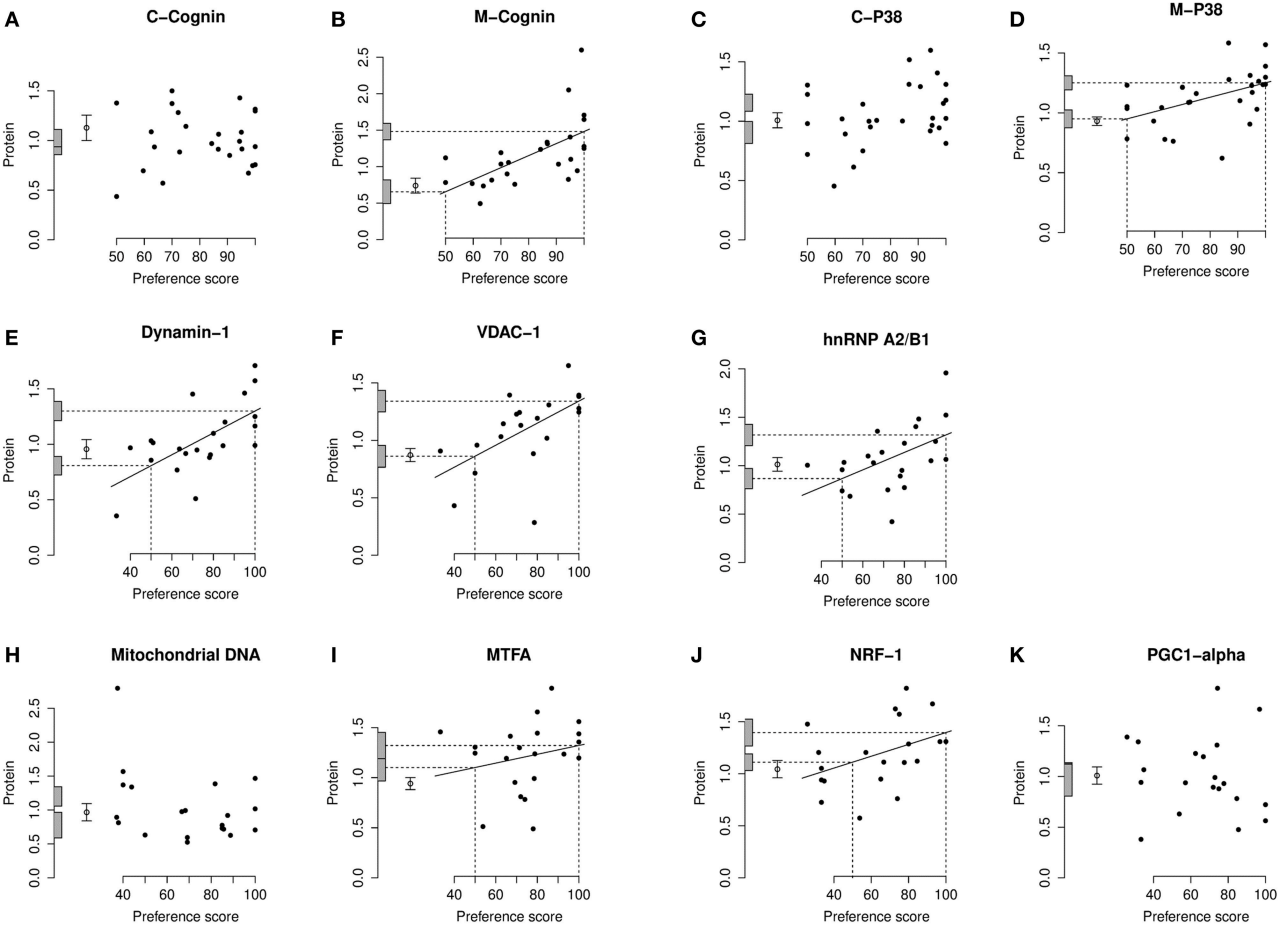
**Left IMM, standardized relative amount of each protein plotted against preference score**. **(A)**, C-cognin; **(B)**, M-cognin; **(C)**, C-P38; **(D)**, M-P38; **(E)**, Dynamin-1; **(F)**, VDAC-1; **(G)**, hnRNP A2/B1; **(H)**, mitochondrial DNA; **(I)**, MTFA; **(J)**, NRF-1; **(K)**, PGC1-α. Each closed circle gives the value for one chick. The least-squares regression line has been drawn only where a regression is significant (*P* < 0.05, two-tailed). The regression line is then interpolated to a preference score of 50 (no preference/learning; left-hand vertical dashed line) and the corresponding amount of protein indicated as an intercept on the ordinate (lower horizontal dashed line). The regression line has also been interpolated to the maximum value of preference score for this experiment (strong preference/learning; right-hand vertical dashed line) and its corresponding amount of protein indicated as an intercept on the ordinate (upper horizontal dashed line). The shaded bars on the ordinate denote +∕− one standard error for each intercept. The open circle and error bars denote the mean value of the untrained chicks +∕− one standard error of the mean.

**Figure 5:**
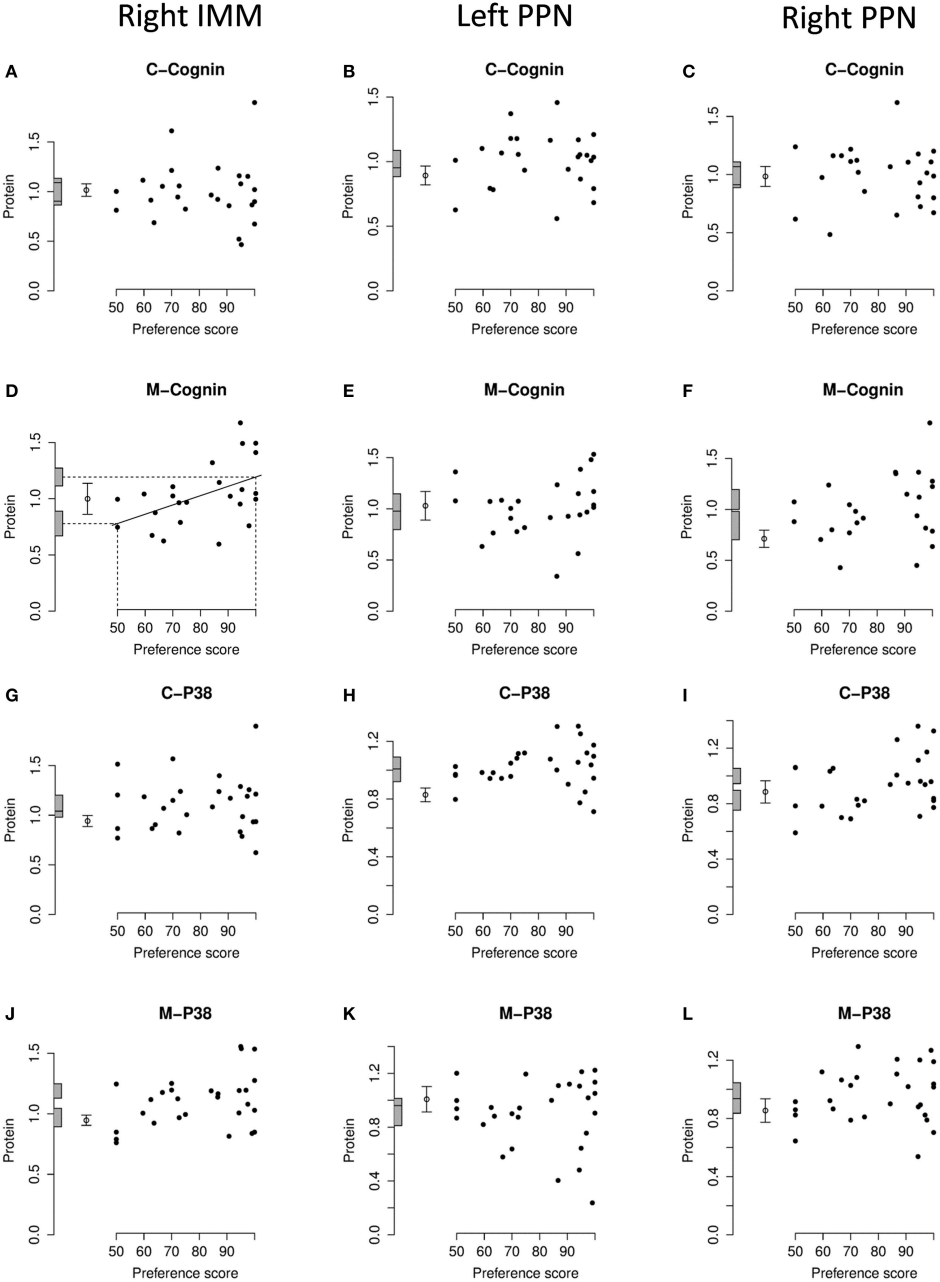
**Right IMM and right and left PPN, standardized relative amount of C- and M-cognin and C- and M-P38, plotted against preference score**. **(A–C)**, C-cognin; **(D–F)**, M-cognin; **(G–I)**, C-P38; **(J–L)**, M-P38. Left-hand column right IMM, middle and right-hand columns left and right PPN, respectively. Conventions otherwise as for Figure 4.

**Figure 6:**
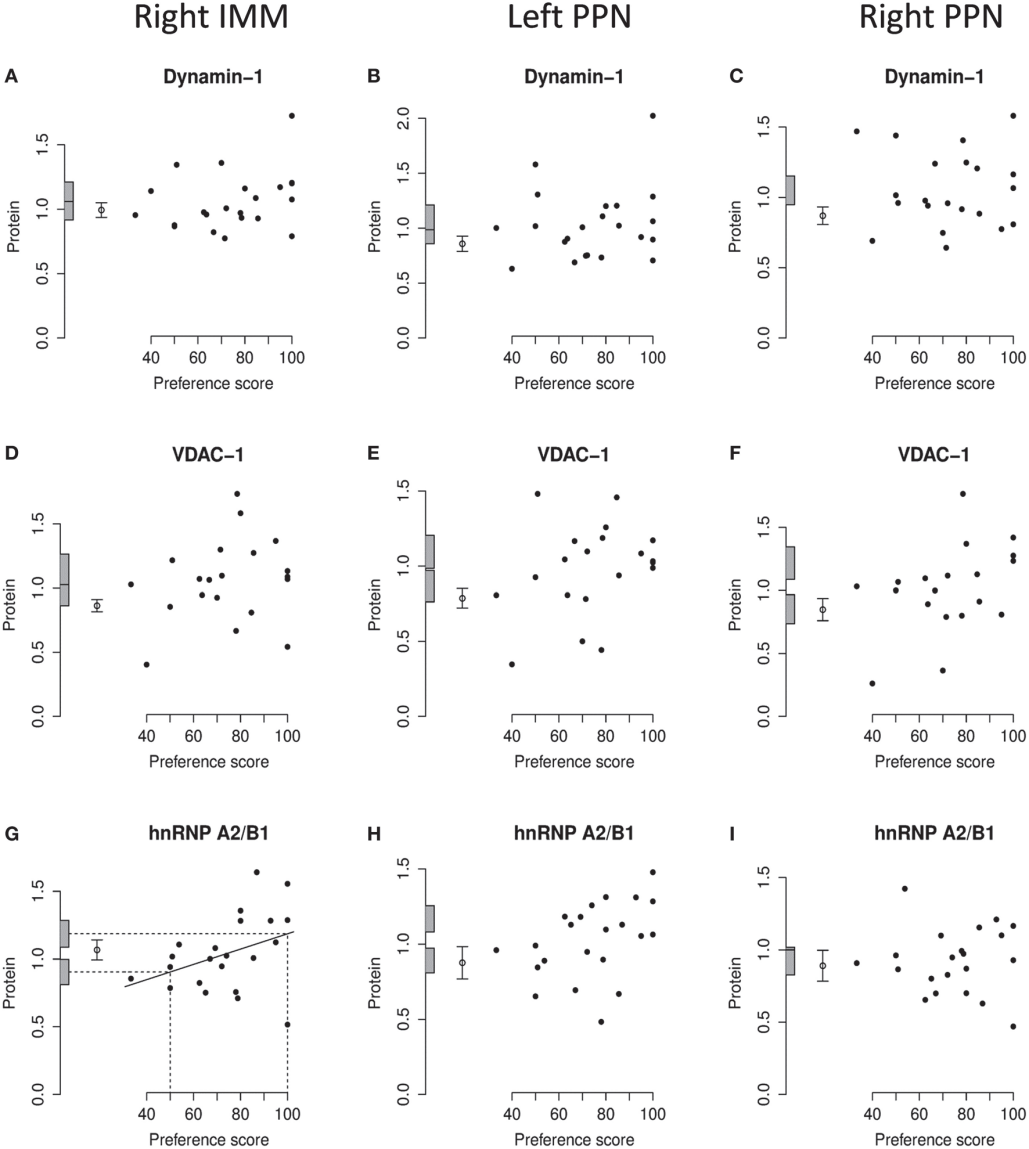
**Right IMM and right and left PPN, standardized relative amount of dynamin-1, VDAC-1, and hnRNP A2/B1, plotted against preference score**. **(A–C)**, dynamin-1; **(D–F)**, VDAC-1; **(G–I)**, hnRNP A2/B1. Left-hand column right IMM, middle and right-hand columns left and right PPN, respectively. Conventions otherwise as for Figure 4.

**Figure 7:**
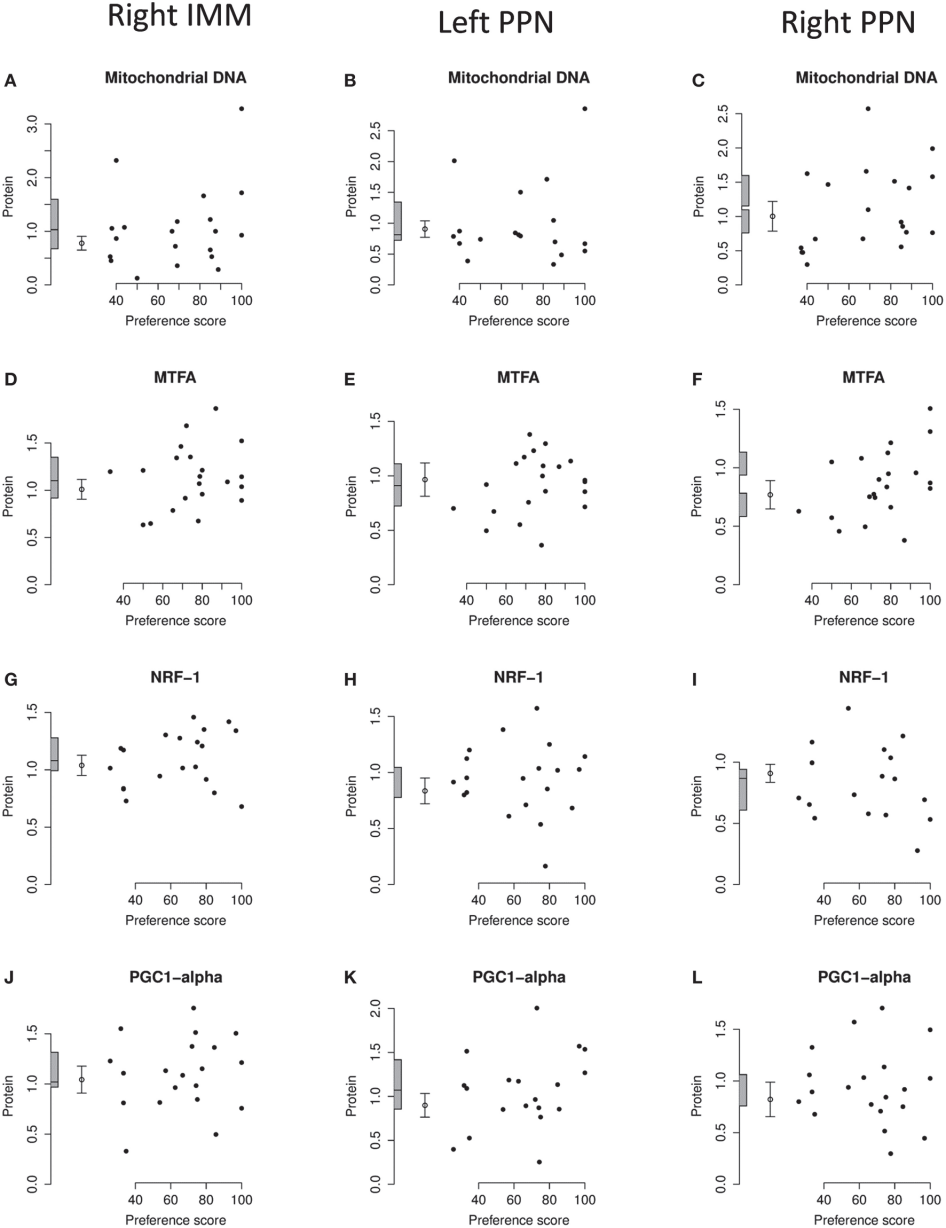
**Right IMM and right and left PPN, standardized relative amounts of mitochondrial copy number and of MTFA, NRF-1, and PGC1-α plotted against preference score**. **(A–C)**, mitochondrial DNA; **(D–F)**, MTFA; **(G–I)**, NRF-1; **(J–L)** PGC1-α. Left-hand column right IMM, middle and right-hand columns left and right PPN, respectively. Conventions otherwise as for Figure 4.

Representative blots are shown in Figure 3. Note that any one blot does not necessarily reflect the quantitative summarized data in Figures 4–**7**.

#### Cognin

Antibodies generated against cognin both in P2 and cytoplasmic fractions reacted with a protein of apparent molecular weight 55–56 kDa, which according to the blocking reaction to immunizing peptide and molecular weight was identified as cognin (Figure 2A).

Cognin resides in two different sub-cellular compartments: (1) endoplasmic reticulum, from which it is suggested to escape to the cell surface after cleavage from its endoplasmic reticulum retention signal and (2) cell surface membrane fraction (Pariser et al., 2000; Capitani and Sallese, 2009). These two subcellular forms may have different functions. Therefore, changes in amounts of cognin were studied separately in cytoplasmic fraction (containing most of the endoplasmic reticulum) and P2 membrane-mitochondrial fraction containing plasma membranes. These two fractions will be referred to as C-cognin and M-cognin, respectively.

##### C-cognin

For C-cognin, no regression with Preference Score, in either IMM or PPN, was significant (Figures 4A, 5A–C).

##### M-cognin

In IMM with data from left and right sides pooled, there was a significant main effect of Preference Score [F_(1, 11)_ = 16.05; P = 0.0021] but no significant interaction between Preference Score and Side (i.e., the slopes of the regressions on left and right IMM did not differ significantly from each other). When the analysis was restricted to left IMM, a significant regression with Preference Score was found [F_(1, 11)_ = 13.01; P = 0.0041]. The slope of this regression was significantly greater than the corresponding slope for C-cognin in left IMM [F_(1, 11)_ = 10.09; P = 0.009]. The intercept of the regression line at preference score 50 did not differ significantly from the untrained value (Figure 4B). That is, preference score had to be substantially greater than the “no preference” value of 50 before a significant increase in M-cognin was observed. Indeed amount of M-cognin predicted for maximum preference score in this experiment (100; strong imprinting) was significantly greater than the mean for untrained chicks [F_(1, 20)_ = 19.81; P = 0.0002]. Residual variance from the regression in trained chicks was greater than that in untrained chicks (although not significantly so). Had it been significantly smaller than the untrained value, there might have been reason to infer that learning had not affected the level of M-cognin (Horn and Johnson, 1989; McCabe and Horn, 1994; McCabe, 2013). The results obtained, however, suggest that an effect of learning arose during training.

Breakdown analysis by side also revealed a significant positive relationship between M-cognin and Preference Score in right IMM [*F*_(1, 10)_ = 6.80; *P* = 0.026; see Figure 5D]. Predicted amount of the protein for preference score 50 was not significantly different from the mean for untrained chicks. However, in contrast to results from the left IMM, the predicted amount of M-cognin for preference score 100 also was not significantly different from the mean for untrained chicks (Figure 5D). That is, even the maximum possible preference score was insufficient for amount of M-cognin in right IMM to be raised above the untrained level. We therefore conclude that the trend in right IMM was not sufficiently strong to justify concluding that a learning-related change had occurred on this side. The residual variance from the regression in right IMM of trained chicks, although lower than the variance in untrained chicks, was not significantly different from the untrained value.

In PPN, regression of the amount of M-cognin with Preference Score was not significant either in the left or the right side (Figures 5E,F).

#### P38

Antibodies against human/mouse P32 protein recognized protein P38 (see Figure 2B), with apparent molecular weight 38 kDa (Okagaki et al., 2000). There is evidence that P32 occurs in cytoplasm and mitochondrial matrix (Dedio and Muller-Esterl, 1996; Muta et al., 1997). P38 was therefore measured in both cytoplasmic and P2 membrane-mitochondrial fractions, as C-P38 and M-P38, respectively.

##### C-P38

There was no significant regression of amount of C-P38 with preference score in any brain region studied (Figures 4C, 5G–I).

##### M-P38

When data from left and right IMM were pooled, there was a significant regression of amount of M-P38 with Preference Score [F_(1, 14)_ = 7.55; P = 0.016]; regression slopes did not differ significantly between sides. A breakdown analysis by side showed there to be a significant regression with Preference Score in left IMM [F_(1, 14)_ = 7.85; P = 0.014; (Figure 4D)]. The mean value in untrained chicks did not differ significantly from the value of the intercept of the regression line at preference score 50, but regression line intercept at preference score 100 was significantly higher than the mean untrained value [F_(1, 23)_ = 21.05; P = 0.00013]. Taken together, the results indicate that there was an increase in M-P38 amount only in chicks showing evidence of learning: despite experience of training stimulus and training wheel, there was no evidence of a change in protein amount unless learning had occurred. The residual variance from the regression analysis was greater than that in untrained chicks, although not significantly so. The fact that the residual variance was not lower than the untrained value indicates that the significant regression was attributable to learning that occurred during training rather than to a predisposition (see Materials and Methods).

The regression in right IMM was not significant (Figure 5J). No regression of M-P38 with Preference Score was found to be significant in left or right PPN (Figures 5K,L).

#### Dynamin-1

Analysis of pooled data from left and right IMM revealed a significant regression with Preference Score [F_[1, 11)_ = 22.90; *P* = 0.0006] and a significant interaction between Preference Score and Side [*F*_(1, 20)_ = 5.04; *P* = 0.036]. Analysis of left and right IMM separately showed that the regression between amount of dynamin-1 and Preference Score was significant only in left IMM [*F*_(1, 11)_ = 17.29; *P* = 0.0016; Figures 4E, 6A]. In left IMM, the intercept corresponding to preference score 50 was lower than the untrained value, but not significantly so. The amount of dynamin-1 corresponding to a preference score of 100 was significantly higher than the mean untrained value [*F*_(1, 21)_ = 7.10; *P* = 0.015]. The residual variance in trained chicks was greater (albeit not significantly) than in untrained chicks. These data indicate that learning, rather than side-effects of training, was responsible for the increased amounts of dynamin-1 in the IMM. In PPN, the regression of amount of dynamin-1 with Preference Score was not significant either in the left or in the right side (Figures 6B,C).

#### VDAC-1

On analysis of data from left and right IMM together, a significant regression with Preference Score was found [*F*_(1, 10)_ = 8.75; *P* = 0.014]. Although there was no significant interaction between Preference Score and Side, analyzing data from the two sides separately showed there to be a significant positive regression between amount of VDAC-1 and Preference Score only in left IMM [*F*_(1, 10)_ = 10.51; *P* = 0.0088; Figures 4F, 6D]. In left IMM, the intercept corresponding to preference score 50 was not significantly different from the untrained value, whereas the intercept corresponding to preference score 100 was significantly higher than the untrained value [*F*_(1, 20)_ = 13.19; *P* = 0.0017]. The residual variance from the regression was greater (although not significantly so) than the untrained value. The results therefore indicate that learning occurring during training was responsible for elevating the amount of VDAC-1 in left IMM. In no other brain region studied was any term significant (Figures 6D–F).

#### hnRNP A2/B1

Analysis of data from left and right IMM together revealed a significant regression with Preference Score [*F*_(1, 11)_ = 8.02; *P* = 0.016] and no significant interaction between Preference Score and Side. Analysis of the data from each side separately showed there to be a significant positive regression of protein amount with Preference Score in left IMM [*F*_(1, 11)_ = 6.70; *P* = 0.025; Figure 4G]. There was also a significant positive regression of hnRNP A2/B1 amount on Preference Score in right IMM [*F*_(1, 11)_ = 5.54; *P* = 0.038; Figure 6G]. For both sides of IMM, the intercept at preference score 50 was not significantly different from the untrained value, and in left IMM, the intercept at preference score 100 was significantly greater than the mean untrained value [*F*_(1, 21)_ = 8.01; *P* = 0.010]. This was not, however, the case in right IMM (Figure 6G). On each side, the residual variance from the regression was greater (but not significantly) than the untrained value. The results indicate that in left IMM, but not right IMM, amount of hnRNP A2/B1 was increased as a consequence of learning.

No regression of hnRNP A2/B1 with Preference Score was found to be significant in left or right PPN (Figures 6H,I).

#### Mitochondrial biogenesis

Of the identified candidate proteins, two of them (VDAC-1 and M-P38) are mitochondrial proteins and their amount was increased with learning in the IMM (see above and also Discussion). Previous research has shown a learning-related increase in the amounts of other mitochondrial proteins in left IMM 24 h after training, namely subunits I and II of cytochrome c oxidase (Solomonia et al., 2011). It is possible that the amounts of these proteins are simply increased due to increase in the number of mitochondria. We have addressed this question by studying changes in the copy number of mitochondrial DNA and in the amounts of MTFA, NRF-1, and PGC1-α, three transcription factors involved in mitochondrial biogenesis (Medeiros, 2008).

#### Mitochondrial DNA

The regression of the amount of mitochondrial-DNA with Preference Score was not significant in any brain region (Figures 4H, 7A–C.)

#### MTFA

Analysis of the data from left and right IMM together revealed no significant regression with Preference Score and no significant interaction between Preference Score and Side. Analysis of data from left and right IMM separately revealed a just-significant regression in left IMM [F_(1, 11)_ = 5.00; P = 0.047; (Figure 4I)], but none in the right (Figure 7D). The amount of MTFA in left IMM at the intercept corresponding to a preference score of 100 was significantly greater than the untrained value [F_(1, 20)_ = 6.97; P = 0.016] and the intercept corresponding to preference score 50 was not significantly different from the untrained value. The residual variance from the regression analysis was greater than that in the untrained chicks, indicating that the increase in amount of MTFA with preference score was attributable to learning that occurred during training. This conclusion should be regarded as tentative, however, in the light of the marginal level of statistical significance in the regression analysis. There was no significant regression in the control brain regions studied (Figures 7E,F).

#### NRF-1

Analysis of data from right and left IMM together showed there to be a significant regression with Preference Score [F_(1, 10)_ = 6.08; P = 0.033]. There was no significant interaction between Preference Score and Side. Analysis of data from left and right IMM separately showed that there was a significant positive correlation between amount of NRF-1 and Preference Score only in left IMM [F_(1, 10)_ = 5.71; P = 0.038; Figures 4J, 7G]. The intercept at preference score 50 was not significantly different from the mean untrained value and the intercept at preference score 100 was significantly higher than the mean in untrained chicks [F_(1, 19)_ = 5.15; P = 0.035]. The residual variance from the regression plot was higher than that in the untrained chicks. Taken together, the data indicate that the increase of the amount of NRF-1 in left IMM with preference score is attributable to learning. No regressions were significant either in left or in right PPN (Figures 7H,I).

#### PGC1-α

There was no significant regression in any of the brain regions studied (Figures 4K, 7J–L).

## Discussion

### Overview

A number of proteins have previously been shown to undergo learning-related regulation in IMM as a result of visual imprinting, either ~1 h or ~24 h after training (see McCabe, 2013; Solomonia and McCabe, 2015 for recent reviews). The present results provide new information about changes in IMM at 24 h, implicate certain mitochondrial proteins and demonstrate a remarkably consistent inter-hemispheric bias in IMM.

The IMM and nearby mesopallial regions play an important role in several types of memory in birds: IMM is involved in passive avoidance learning (Rose, 2000; Gibbs and Summers, 2002); anterior medial mesopallium has been implicated in auditory imprinting in domestic chicks (Bredenkötter and Braun, 1997) and caudal medial mesopallium of songbirds in storing information about a song that has been learned (Bolhuis and Gahr, 2006; Gobes et al., 2010).

Criteria for inferring that a change in protein level is learning-related following imprinting are given in Materials and Methods (Section Statistical Analysis of Immunoblotting and DNA Data). Previous work using similar criteria (reviewed in Horn, 2004; McCabe, 2013; Solomonia and McCabe, 2015) has indicated that learning-related changes at 24 h after training are predominantly expressed in left IMM. Changes often also occur in right IMM at 24 h, but in general are less clearly expressed. This trend, of predominance of the effect in left IMM, is clear in the present study. Of the learning-related changes found, namely in M-cognin, M-P38, dynamin-1, VDAC-1, hnRNP A2/B1, MTFA, and NRF-1, only dynamin-1 showed a significant interaction between Preference Score and Side, i.e., an inter-hemispheric difference in slope of the regression. However, when the data were analyzed separately for each side, all of the proteins showed a learning-related change in left IMM. Significant regressions were found in right IMM for M-cognin and hnRNP A2/B1, but on this side, the levels of these proteins corresponding to the maximum possible preference score were not significantly different from the mean value for untrained chicks. Thus, all of the learning-related effects were stronger in left IMM.

It has long been known that the IMM expresses a functional asymmetry following imprinting (Horn, 1985) and passive avoidance learning (Rose, 2000). The increase in length of the postsynaptic density of axospinous synapses following imprinting training is restricted to the left IMM (Bradley et al., 1981; Horn et al., 1985), as is the learning-related increase in number of NMDA receptors (McCabe and Horn, 1988). Johnston et al. (1995) and Johnston and Rogers (1996, 1998) have also found asymmetries of NMDA glutamate receptor function in MM in relation to imprinting. Lesion studies indicate that, whereas both left and the right IMM can support both acquisition and retention of a preference by imprinting (McCabe et al., 1981, 1982; Horn et al., 1983), left and right IMM behave differently: right IMM is necessary for memory in a region S' outside the IMM. Moreover, if right IMM is ablated ≤3 h after training, retention depends critically on the left IMM (Cipolla-Neto et al., 1982). The left IMM is distinctive in displaying a strong correlation between levels of subunits CO-I and CO-II of cytochrome c oxidase, an enzyme that is critical for oxidative phosphorylation; right IMM shows no such relationship. In reporting this asymmetry, Solomonia et al. (2011) suggested that left IMM is specialized at the start of the sensitive period for imprinting, possibly by precocial development, for the efficient acquisition and processing of information acquired through imprinting. Moorman and Nicol (2015) discuss electrophysiological evidence for hemispheric asymmetry in the IMM. Biochemical, anatomical, electrophysiological, and functional asymmetry having been clearly established, the present results indicate that that the contribution of left IMM to memory is associated with higher levels of specific proteins compared to right IMM.

### Cognin

Cognin initially was considered as a chick retina-specific cell adhesion molecule (Hausman and Moscona, 1976). That cognin is a protein disulphide isomerase (PDI) was recognized later (Pariser et al., 2000). PDI could facilitate the proper folding of nascent proteins and be involved in refolding of partially denatured proteins (Hatahet and Ruddock, 2009; Kozlov et al., 2010). It seems likely to be important for the maintenance of normal neural function: altered PDI activity has been linked to a number of neurodegenerative diseases including Alzheimer's disease, Parkinson's disease, Huntington's disease, and amyotrophic lateral sclerosis (Andreu et al., 2012). PDI is a target of cyclopentenone prostaglandins (Liu et al., 2015). These highly reactive molecules are downstream mediators of cyclooxygenase-2 toxicity during ischemic brain injury and overexpression of PDI is protective against brain ischemic injury (Tanaka et al., 2000; Liu et al., 2015).

As mentioned in Results, cognin could reside in two subcellular compartments (endoplasmic reticulum and plasma membrane) and therefore we have studied its changes in corresponding tissue fractions. The results revealed learning-related changes in M-cognin but not C-cognin, in left IMM. The slopes of the regressions differ significantly between C- and M-cognin in left IMM. The effect is thus restricted to the membrane-mitochondrial fraction. Two hypotheses, not mutually exclusive, could explain the effect: (i) the amount of cognin is increased in the part of the endoplasmic reticulum associated with the P2 membrane-mitochondrial fraction; (ii) increased translocation of C-cognin to the plasma membrane, where it could be involved in the modulation of cell adhesion and contribute to the stability of membrane proteins. This may occur in conjunction with up-regulation of neural cell adhesion molecules in IMM, shown to be present 24 h after training by Solomonia et al. (1998).

There was a significant regression of M-cognin with Preference Score in the IMM. At 24 h after training, significant regressions were previously found to be restricted to left IMM (for recent reviews see McCabe, 2013; Solomonia and McCabe, 2015). In spite of the significant regression in right IMM, the predicted amount of M-cognin for preference score 100 was not significantly different from the mean value for untrained chicks; that is, the maximum preference score was insufficient to raise the amount of M-cognin in right IMM above the untrained level. There is strong evidence that both sides of IMM are memory stores for features of the imprinting stimulus. However, right IMM has an additional role linked with the formation of the so-called S' memory store outside IMM (for review see Horn, 2004). It is possible that the two parallel processes—one linked with memory storage and the other with the establishment of S', partially mask learning-related changes in M-cognin in right IMM.

As far as we know, our results are the first implicating cognin in memory.

### P38

The amount of M-P38 was increased in a learning-related manner in the IMM but not in the other brain regions studied. No significant changes were found in any brain region for C-P38.

The mitochondrial matrix is one of the intracellular sites where P38/P32 is found (Muta et al., 1997). Various functions have been assigned to mitochondrial P32. This protein can regulate mitochondrial morphology and dynamics by promoting parkin degradation through autophagy (Li et al., 2011). The neural processes that are engaged in learning and memory, and which are particularly clear in the IMM, may place a heavy demand on oxidative metabolism (Gibbs et al., 2006). P32 is involved in mitochondrial translational processes (Uchiumi and Kang, 2012). P32 knockdown in human cancer cells inhibits the synthesis of the mitochondrial DNA-encoded proteins CO-I and CO-II (Fogal et al., 2010). The amounts of CO-I and CO-II are increased in a learning-related way in leftIMM 24 h after training (Solomonia et al., 2011) and these changes could therefore be influenced by M-P38.

Our results are, we believe, the first indicating that M-P38 is involved in memory.

### Dynamin-1

A learning-related increase in the amount of dynamin-1 was found in left IMM of the trained chicks and not in the other regions studied.

In the process of synaptic vesicle recycling dynamin, a GTPase, is involved in membrane fission and clathrin lattice disassembly (for review see Saheki and De Camilli, 2012). Dynamin is encoded by three genes—DNM1, DNM2, and DNM3. The brain is characterized by containing the highest amount of the corresponding proteins as compared to other tissues (Ferguson et al., 2007). Dynamin-1 and dynamin-3 are strongly expressed in neurons, the level of expression of dynamin-1 being much the higher of the two (Cao et al., 1998; Gray et al., 2003; Ferguson et al., 2007). Dynamin-2 is expressed in all tissues (Cao et al., 1998; Ferguson et al., 2007). It has been suggested that dynamin-1 is selectively implicated in synaptic vesicle recycling, dynamin-3 in endocytosis within dendritic spines and excitatory neurotransmitter receptor trafficking, and dynamin 2 in a range of maintenance functions (Gray et al., 2003; Lu et al., 2007).

We have previously reported a learning-related increase of clathrin heavy chain at 24 h (but not 9 h) after training, in left IMM but not right IMM or control brain regions (Solomonia et al., 1997). That result raised the possibility that the turnover and/or number of synaptic vesicles in axon terminals and neurotransmitter release in left IMM are increased 24 h after training (Solomonia et al., 1997; for review see Solomonia and McCabe, 2015). Since dynamin-1 is involved in synaptic vesicle recycling, our results suggest that both dynamin-1 and clathrin heavy chain contribute to memory formation by presynaptic modulation of neurotransmitter release.

### VDAC-1

The mitochondria of all eukaryotic cells contain the mitochondrial permeability transition pore (MPTP), which is formed from three proteins: VDAC, the adenine nucleotide transporter and cyclophilin D. Besides apoptosis, MPTP and its components have been implicated in synaptic calcium buffering by mitochondria, and in synaptic plasticity and learning in mice (Weeber et al., 2002; Levy et al., 2003). In VDAC-1-deficient mice, fear conditioning and spatial learning are disrupted (Weeber et al., 2002).

Our 2-D electrophoresis experiments identified VDAC-1 as a protein which was up-regulated in the left IMM in a learning-related manner. The changes in VDAC-1 reported here, along with the changes in M-P38 and previously reported learning-related changes in CO-I and CO-II (Solomonia et al., 2011) suggest that the mitochondrial proteome in the left IMM has an important role to play in memory for the imprinting stimulus.

### hnRNP A2/B1

As for the other proteins studied, the main region associated with the learning-related changes for the hnRNP A2/B1 was left IMM. In right IMM, the regression of the amount of hnRNP A2/B1 with Preference Score was also significant. However, the amount of the protein corresponding to the intercept of preference score 100 was not significantly different from the mean amount in untrained chicks; despite the significant regression, the maximum preference score attainable was insufficient to raise the level of hnRNP A2/B1 above the untrained value.

Synaptic plasticity can require activity-dependent transport and translation of dendritic mRNA, with concomitant alterations in local proteins (for review see Sutton and Schuman, 2006). hnRNP A2/B1 is an RNA-binding protein involved in mRNA trafficking. Neuronal activity induces synaptic delivery of hnRNP A2/B1 by a BDNF-dependent mechanism in cultured hippocampal neurons (Leal et al., 2014). hnRNP A2/B1 recognizes a cis-acting element present in myelin basic protein mRNA. Targeting of mRNAs by this element in neurons is involved in the delivery to dendrites of a number of proteins including α-calcium/calmodulin-dependent protein kinase II (α-CaMKII) (Ainger et al., 1997; Munro et al., 1999; Gao et al., 2008). α-CaMKII is elevated in the IMM in a learning-related manner in the IMM 1 h after training, but not at the 24 h time-point chosen for the present study (Solomonia et al., 2005). hnRNP A2/B1 may nevertheless influence the delivery of this important enzyme to dendritic locations and modulate the delivery and translation of memory-related mRNAs in the IMM.

Defects in hnRNP A2/B1 could give rise to certain neurological disorders. This protein possesses a prion-like domain, mutation of which causes multisystem proteinopathy and amyotrophic lateral sclerosis (Kim et al., 2013). The prion-like domain is of further interest in view of the fact that the prion-like aggregation of cytoplasmic polyadenylation element-binding protein 3 (CPEB3) has been implicated in synaptic plasticity and memory in mice (Fioriti et al., 2015).

### Mitochondrial biogenesis

The present results reveal learning-related increases in the amounts of the mitochondrial proteins M-P38 and VDAC-1 in left IMM of the trained chicks. Previous results (Solomonia et al., 2011) have shown learning-related changes in CO-I and CO-II, two subunits of cytochrome c oxidase, also in left IMM 24 h after training. Cytochrome c oxidase comprises 13 subunits; 3 of them (including CO-I and CO-II) are encoded by the mitochondrial genome and are thus among the rare examples of bi-genomic proteins. The coordinated regulation of such a multi-subunit, multi-chromosomal, bi-genomic enzyme poses an especial challenge for neurons, whose mitochondria are widely distributed in extensive dendritic and axonal processes (for review see Wong-Riley, 2012). Using biochemical methods, we have enquired whether the learning-related increase in mitochondrial proteins encoded both by mitochondrial and nuclear genomes are the consequence of the increased mtDNA copy number. No change was found, suggesting stability of the mitochondrial genome despite changes in amounts of mitochondrial-encoded proteins. This result does not exclude the possibility of mitochondrial fusion, since it is known that fusion can occur as protection against neurodegeneration in cerebellum (Chen et al., 2007, 2010). Since no significant changes in amount of mtDNA were found, despite clear learning-related changes in amounts of mitochondrial proteins in the same brain region, we conclude that that the changes in protein level reflect increased transcription of mtDNA.

We have also studied transcription factors implicated in mitochondrial biogenesis. It should be noted that for none of them is this their sole function.

For MTFA, a learning-related change was observed only in left IMM of trained chicks. MTFA is a transcription factor produced in the cytoplasm and imported into mitochondria. In mitochondria, MTFA controls the expression of mtDNA-encoded genes and mtDNA replication (Fisher and Clayton, 1988; Ekstrand et al., 2004; Scarpulla, 2006, 2008; Shadel, 2008). Replication of mtDNA coincides with transcription in time and space, and collision between the transcription and replication machineries is inevitable (Pomerantz and O'Donnell, 2010; Agaronyan et al., 2015). Thus, the learning-related increase in MTFA level could be involved either in mtDNA replication or increased transcription. As the mtDNA amount is not increased with learning and at the same time the amount of mitochondrially encoded proteins are increased (Solomonia et al., 2011) we propose that increase in the amounts of MTFA is linked with increased mitochondrial transcription in left IMM during imprinting.

Learning-related changes were also observed for NRF-1. This transcription factor was discovered as a regulator of somatic cytochrome c, the substrate for cytochrome c oxidase (for review see Wong-Riley, 2012). The level of NRF-1 mRNA as well as protein responds to changes in neuronal activity. Existing data strongly indicate that NRF-1 directly regulates the expression of 10 nuclear-encoded subunits of cytochrome c oxidase holoenzyme and, indirectly, expression of the three mitochondrially-encoded subunits. In this indirect regulation, NRF-1 acts in association with MTFA (Wong-Riley, 2012). Thus, the targets of NRF-1 action are consistent with the learning-related increase in CO-I and CO-II subunits in the IMM (Solomonia et al., 2011). NRF-1 also regulates the expression of NMDA receptor subunits (Priya et al., 2013) and sodium/potassium ATPase (Johar et al., 2012). These findings implicate NRF-1 in the tight coupling of neuronal activity, energy generation, and energy consumption (Johar et al., 2012; Wong-Riley, 2012; Priya et al., 2013).

No significant changes with learning were observed for PGC-1α. This transcriptional co-activator does not bind directly to DNA, but rather responds to appropriate signals in a tissue-specific manner, and interacts with nuclear receptors and transcription factors to activate genes involved in energy and nutrient homeostasis; PGC-1α is also involved in mitochondrial biogenesis (Puigserver et al., 1998; Lehman et al., 2000; reviewed in Wong-Riley, 2012). NRF-1 is one of the transcription factors with which it interacts.

### Conclusions

Proteomic studies of chick brain regions involved in the learning and memory of visual imprinting, and of control brain regions 24 h after training have revealed new and interesting molecular features. Our results indicate that the biochemical processes involved in learning and memory cover a wide range of cellular activities, including stabilization of protein structures, increased mRNA trafficking, synaptic vesicle recycling and specific changes in the mitochondrial proteome.

## Funding

BBSRC grants 8/S18043, BB/H018948/1, Isaac Newton Trust (McCabe). S. Rustaveli National Science Foundation grant 31/01; Ilia State University (Solomonia).

### Conflict of interest statement

The authors declare that the research was conducted in the absence of any commercial or financial relationships that could be construed as a potential conflict of interest.
