# Supplementary material for: A Proteomic Study of Memory After Imprinting in the Domestic Chick
Source: Front Behav Neurosci. 2015 Nov 26;9:319. doi: 10.3389/fnbeh.2015.00319 (PMC4660867; doi:10.3389/fnbeh.2015.00319)
Supplement: Supplementary file 1 [file DataSheet1.DOCX]

**
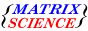
Mascot Search Results**

**User : Mascot Daemon**

**Email :**

**Search title : Submitted from 6010 tony LTQ params by Mascot Daemon on MINIPROTEOME**

**MS data file : C:\Documents and Settings\svh24.PROTEOMICS\Desktop\Svenja Mascot Files\plate6010tony_20101223\6010wa5s270.mgf**

**Database : chicken10 REFSEQ_082010 (19127 sequences; 7388293 residues)**

**Timestamp : 5 Jan 2011 at 11:46:19 GMT**

| **Protein hits    :** | [**gi\|21703694\|gb\|AAA49054.2\|**](http://192.168.4.110/mascot/cgi/master_results.pl?file=..%2Fdata%2F20110105%2FF050944.dat&REPTYPE=peptide&_sigthreshold=0.05&REPORT=AUTO&_server_mudpit_switch=99999999&_ignoreionsscorebelow=20&_showsubsets=0&_showpopups=TRUE&_sortunassigned=scoredown&_requireboldred=0#Hit1) | cognin/prolyl-4-hydroxylase/protein disulfide isomerase [Gallus gallus] |
| --- | --- | --- |

**Probability Based Mowse Score**

Ions score is -10*Log(P), where P is the probability that the observed match is a random event.
Individual ions scores > 31 indicate identity or extensive homology (p<0.05).
Protein scores are derived from ions scores as a non-probabilistic basis for ranking protein hits.


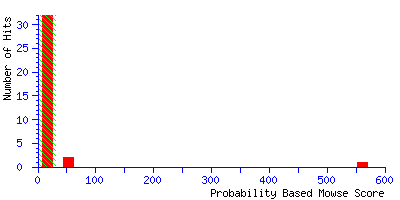


Top of Form

**Peptide Summary Report**

|  |  |  | [Help](http://192.168.4.110/mascot/help/results_help.html#FORMAT) |
| --- | --- | --- | --- |
|  | Significance threshold p<  | Max. number of hits  |  |
|  | Standard scoring  MudPIT scoring  | Ions score or expect cut-off  | Show sub-sets  |
|  | Show pop-ups  Suppress pop-ups  | Sort unassigned  | Require bold red  |

Bottom of Form

Top of Form

        **Error tolerant**

| **1.** | [gi\|21703694\|gb\|AAA49054.2\|](http://192.168.4.110/mascot/cgi/protein_view.pl?file=../data/20110105/F050944.dat&hit=gi%7c21703694%7cgb%7cAAA49054%2e2%7c&px=1&_server_mudpit_switch=99999999&_ignoreionsscorebelow=20)    **Mass:** 58896    **Score:** 561    **Queries matched:** 12   **emPAI:** 0.46 |
| --- | --- |
|  | cognin/prolyl-4-hydroxylase/protein disulfide isomerase [Gallus gallus] |

|  | Check to include this hit in error tolerant search or archive report |
| --- | --- |
|  |  |

|  | **Query** | **Observed** | **Mr(expt)** | **Mr(calc)** | **Delta** | **Miss** | **Score** | **Expect** | **Rank** | **Peptide** |
| --- | --- | --- | --- | --- | --- | --- | --- | --- | --- | --- |
|  | [75](http://192.168.4.110/mascot/cgi/peptide_view.pl?file=../data/20110105/F050944.dat&query=75&hit=1&index=gi%7c21703694%7cgb%7cAAA49054%2e2%7c&px=1&section=5) | **519.1592** | **1036.3038** | **1036.4825** | **-0.1787** | **0** | **35** | **0.028** | **1** | **R.NNFEGDLTK.D** |
|  | [125](http://192.168.4.110/mascot/cgi/peptide_view.pl?file=../data/20110105/F050944.dat&query=125&hit=1&index=gi%7c21703694%7cgb%7cAAA49054%2e2%7c&px=1&section=5) | **603.7658** | **1205.5171** | **1205.6213** | **-0.1042** | **0** | **65** | **2.7e-05** | **1** | **R.LITLEEEMTK.Y** |
|  | [136](http://192.168.4.110/mascot/cgi/peptide_view.pl?file=../data/20110105/F050944.dat&query=136&hit=1&index=gi%7c21703694%7cgb%7cAAA49054%2e2%7c&px=1&section=5) | **647.1967** | **1292.3789** | **1292.5918** | **-0.2129** | **0** | **82** | **6e-07** | **1** | **K.MDSTANEVEAVK.I** |
|  | [142](http://192.168.4.110/mascot/cgi/peptide_view.pl?file=../data/20110105/F050944.dat&query=142&hit=1&index=gi%7c21703694%7cgb%7cAAA49054%2e2%7c&px=1&section=5) | **671.1600** | **1340.3054** | **1340.5884** | **-0.2830** | **0** | **(51)** | **0.00054** | **1** | **K.NFEEVAFDENK.N** |
|  | [143](http://192.168.4.110/mascot/cgi/peptide_view.pl?file=../data/20110105/F050944.dat&query=143&hit=1&index=gi%7c21703694%7cgb%7cAAA49054%2e2%7c&px=1&section=5) | **671.2400** | **1340.4654** | **1340.5884** | **-0.1230** | **0** | **(69)** | **9.8e-06** | **1** | **K.NFEEVAFDENK.N** |
|  | [144](http://192.168.4.110/mascot/cgi/peptide_view.pl?file=../data/20110105/F050944.dat&query=144&hit=1&index=gi%7c21703694%7cgb%7cAAA49054%2e2%7c&px=1&section=5) | **671.2609** | **1340.5072** | **1340.5884** | **-0.0813** | **0** | **72** | **4.8e-06** | **1** | **K.NFEEVAFDENK.N** |
|  | [150](http://192.168.4.110/mascot/cgi/peptide_view.pl?file=../data/20110105/F050944.dat&query=150&hit=1&index=gi%7c21703694%7cgb%7cAAA49054%2e2%7c&px=1&section=5) | **698.8169** | **1395.6192** | **1395.7398** | **-0.1206** | **0** | **67** | **1.7e-05** | **1** | **K.YQLSQDGVVLFK.K** |
|  | [151](http://192.168.4.110/mascot/cgi/peptide_view.pl?file=../data/20110105/F050944.dat&query=151&hit=1&index=gi%7c21703694%7cgb%7cAAA49054%2e2%7c&px=1&section=5) | **698.8633** | **1395.7121** | **1395.7398** | **-0.0277** | **0** | **(62)** | **5.1e-05** | **1** | **K.YQLSQDGVVLFK.K** |
|  | [224](http://192.168.4.110/mascot/cgi/peptide_view.pl?file=../data/20110105/F050944.dat&query=224&hit=1&index=gi%7c21703694%7cgb%7cAAA49054%2e2%7c&px=1&section=5) | **957.9502** | **1913.8858** | **1914.0098** | **-0.1240** | **0** | **118** | **1.4e-10** | **1** | **K.SNQLPLVIEFTEQTAPK.I** |
|  | [399](http://192.168.4.110/mascot/cgi/peptide_view.pl?file=../data/20110105/F050944.dat&query=399&hit=1&index=gi%7c21703694%7cgb%7cAAA49054%2e2%7c&px=1&section=5) | **1322.5029** | **2642.9913** | **2643.2956** | **-0.3043** | **0** | **(109)** | **8e-10** | **1** | **K.EFLLAAESVDDIPFGISSSADVFSK.Y** |
|  | [400](http://192.168.4.110/mascot/cgi/peptide_view.pl?file=../data/20110105/F050944.dat&query=400&hit=1&index=gi%7c21703694%7cgb%7cAAA49054%2e2%7c&px=1&section=5) | **1322.9600** | **2643.9054** | **2643.2956** | **0.6099** | **0** | **123** | **3.2e-11** | **1** | **K.EFLLAAESVDDIPFGISSSADVFSK.Y** |
|  | [401](http://192.168.4.110/mascot/cgi/peptide_view.pl?file=../data/20110105/F050944.dat&query=401&hit=1&index=gi%7c21703694%7cgb%7cAAA49054%2e2%7c&px=1&section=5) | **1323.0000** | **2643.9854** | **2643.2956** | **0.6899** | **0** | **(97)** | **1.4e-08** | **1** | **K.EFLLAAESVDDIPFGISSSADVFSK.Y** |

|  | |
| --- | --- |
|  | **Proteins matching the same set of peptides:** |

|  | [gi\|118099885\|ref\|XP_420095.2\|](http://192.168.4.110/mascot/cgi/protein_view.pl?file=../data/20110105/F050944.dat&hit=gi%7c118099885%7cref%7cXP_420095%2e2%7c&px=1&_server_mudpit_switch=99999999&_ignoreionsscorebelow=20)    **Mass:** 45380    **Score:** 561    **Queries matched:** 12  PREDICTED: similar to cognin/prolyl-4-hydroxylase/protein disulfide isomerase [Gallus gallus] |
| --- | --- |
