# Supplementary material for: A Proteomic Study of Memory After Imprinting in the Domestic Chick
Source: Front Behav Neurosci. 2015 Nov 26;9:319. doi: 10.3389/fnbeh.2015.00319 (PMC4660867; doi:10.3389/fnbeh.2015.00319)
Supplement: Supplementary file 2 [file DataSheet2.DOCX]

**
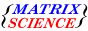
Mascot Search Results**

**User : Mascot Daemon**

**Email :**

**Search title : Submitted from 6010 tony LTQ params by Mascot Daemon on MINIPROTEOME**

**MS data file : C:\Documents and Settings\svh24.PROTEOMICS\Desktop\Svenja Mascot Files\plate6010tony_20101223\6010wb5s117.mgf**

**Database : chicken10 REFSEQ_082010 (19127 sequences; 7388293 residues)**

**Timestamp : 5 Jan 2011 at 11:46:52 GMT**

| **Protein hits    :** | [**gi\|118100069\|ref\|XP_415748.2\|**](http://192.168.4.110/mascot/cgi/master_results.pl?file=..%2Fdata%2F20110105%2FF050945.dat&REPTYPE=peptide&_sigthreshold=0.05&REPORT=AUTO&_server_mudpit_switch=99999999&_ignoreionsscorebelow=20&_showsubsets=0&_showpopups=TRUE&_sortunassigned=scoredown&_requireboldred=0#Hit1) | PREDICTED: similar to p32 subunit of splicing factor SF2 [Gallus gallus] |
| --- | --- | --- |
|  |  |  |
|  |  |  |
|  |  |  |
|  |  |  |
|  |  |  |

**Probability Based Mowse Score**

Ions score is -10*Log(P), where P is the probability that the observed match is a random event.
Individual ions scores > 31 indicate identity or extensive homology (p<0.05).
Protein scores are derived from ions scores as a non-probabilistic basis for ranking protein hits.


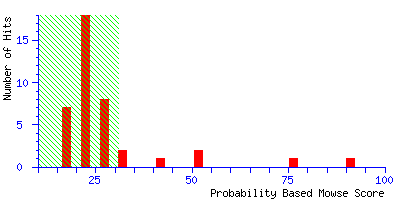


Top of Form

**Peptide Summary Report**

|  |  |  | [Help](http://192.168.4.110/mascot/help/results_help.html#FORMAT) |
| --- | --- | --- | --- |
|  | Significance threshold p<  | Max. number of hits  |  |
|  | Standard scoring  MudPIT scoring  | Ions score or expect cut-off  | Show sub-sets  |
|  | Show pop-ups  Suppress pop-ups  | Sort unassigned  | Require bold red  |

Bottom of Form

Top of Form

        **Error tolerant**

| **1.** | [gi\|118100069\|ref\|XP_415748.2\|](http://192.168.4.110/mascot/cgi/protein_view.pl?file=../data/20110105/F050945.dat&hit=gi%7c118100069%7cref%7cXP_415748%2e2%7c&px=1&_server_mudpit_switch=99999999&_ignoreionsscorebelow=20)    **Mass:** 27702    **Score:** 91     **Queries matched:** 2   **emPAI:** 0.12 |
| --- | --- |
|  | PREDICTED: similar to p32 subunit of splicing factor SF2 [Gallus gallus] |

|  | Check to include this hit in error tolerant search or archive report |
| --- | --- |
|  |  |

|  | **Query** | **Observed** | **Mr(expt)** | **Mr(calc)** | **Delta** | **Miss** | **Score** | **Expect** | **Rank** | **Peptide** |
| --- | --- | --- | --- | --- | --- | --- | --- | --- | --- | --- |
|  | [2](http://192.168.4.110/mascot/cgi/peptide_view.pl?file=../data/20110105/F050945.dat&query=2&hit=1&index=gi%7c118100069%7cref%7cXP_415748%2e2%7c&px=1&section=5) | **382.7226** | **763.4306** | **763.4116** | **0.0190** | **0** | **31** | **0.092** | **1** | **K.FLEDLK.S** |
|  | [116](http://192.168.4.110/mascot/cgi/peptide_view.pl?file=../data/20110105/F050945.dat&query=116&hit=1&index=gi%7c118100069%7cref%7cXP_415748%2e2%7c&px=1&section=5) | **755.3200** | **1508.6254** | **1508.6783** | **-0.0529** | **0** | **60** | **8.9e-05** | **1** | **R.EVSFQPTGESDWK.D** |
